# EARLY DETECTION: A COMPREHENSIVE COGNITIVE SCREENING TOOL FOR ADRD THROUGH INNOVATIVE MOBILE APP TECHNOLOGY

**DOI:** 10.1093/geroni/igae098.3955

**Published:** 2024-12-31

**Authors:** Lenora Smith, Charles O’Brien

**Affiliations:** University of Alabama in Huntsville, Huntsville, Alabama, United States; University of Alabama in Huntsville, Huntsville, Alabama, United States

## Abstract

Currently, there are no curative treatments for Alzheimer’s Disease; it is imperative to detect cognitive decline early to provide lifestyle factors that may play a protective role in older individuals, delaying the progression of mild cognitive impairment (MCI). Neurospective is a mobile application platform being designed to facilitate the early detection of cognitive decline, including MCI and pre-clinical Alzheimer’s disease and related dementias (ADRD). It will utilize a unique combination of engaging cognitive assessment games, diverse user data collection, and machine learning to establish a robust and accessible screening tool. The app is being built around cognitive assessments from a suite of mini-games. The activities are inspired by established screening tests, like 3MS, MoCA, and SLUMS, to assess language, problem-solving, reasoning, memory, and motor function. This innovative app will be easily accessible for screening MCI and assess outcomes based on an evolving machine learning algorithm. The continuously evolving models, based on user data, will lead to increasingly accurate predictions of cognitive health and potential ADRD risk. This app will initially establish a relative measure of cognitive health within the population, eventually progressing into a reliable screening tool for early detection of MCI. Neurospective presents a unique and promising approach to early detection of MCI through its accessible mobile app format, comprehensive data collection, and evolving machine learning models. This project has the potential to significantly improve our understanding of cognitive health and contribute to earlier intervention for individuals at risk of ADRD.

